# Eight Months’ Constipation

**Published:** 1874-11

**Authors:** Thomas D. Strong

**Affiliations:** Westfield, N. Y.


Case of Habitual and Excessive Constipation; Eight Months and Sixteen Days between Fecal Evacuations—*By Thomas D. Strong, M.D., of Westfield, N. Y.*—M. B., now residing in Sheridan, Chautauqua county, N. Y., æt. 26, height 5 feet 10 inches; unloaded weight 125; skin pale, and has a waxy look; tongue clean and pale; has been habitually constipated from childhood.

The first medical history of him which I have obtained is by Dr. George S. Harrison, of Sinclearville, who attended Brooks when two years old for costiveness. His habit then was to go about two weeks without fecal evacuation.

Several years later he attended him for diphtheria, and the time was then extended to six weeks. Dr. H. has occasionally seen him, and watched the case to this time, and says the disease has been gradually and steadily increasing. For the past six years he has been under my observation. * * *

Dr. G. B. Bishop, of Silver Creek, who is now B.'s medical adviser, wrote me May 7, 1874: "His bowels have moved a little several times since you saw him at my house, March 14."

Once or twice a year he has an attack of vomiting, attended with violent pain in stomach and bowels. In 1872 he had such an attack, which was controlled by hypodermic injection of morphia; and again, in 1873, the same occurred, and the bowels were completely evacuated.

The process of defecation lasts two to four days, at which time he is sick, and becomes much exhausted.

The dejections look like brown paper chewed (paper wads of school-boy days.)

There is free escape of gas per anum.

The weight of fecal matter at one dejection was approximately obtained. He was accidentally weighed just before the movement, and again as soon as he could get to the scales. The difference was forty pounds.

The longest interval between any fecal discharges occurred four years since, and was *eight months and sixteen days.*

He is a laborer, and does considerable light work on a farm. His abdomen, when loaded, is hard; the diaphragm crowded high in the chest; the colon immensely distended, and traceable like a huge sausage.

He has been under the care of many physicians of all kinds, both intelligent and otherwise, and every imaginable treatment, followed by no permanent benefit.

P. S.—Since writing the above report, Dr. Bishop writes me that he has pursued a course agreed upon by us last spring, and has given to B. the elix. cinchona, iron and strychnia, with occasional small doses of calomel. The result has been evacuations at short intervals, so that he has diminished in size materially, and his general health is improved.

